# Secondary rotational atherectomy is associated with reduced occurrence of prolonged ST-segment elevation following ablation

**DOI:** 10.1007/s11739-023-03385-7

**Published:** 2023-08-11

**Authors:** Daisuke Kanda, Takuro Takumi, Ryo Arikawa, Kazuhiro Anzaki, Takeshi Sonoda, Kenta Ohmure, Daichi Fukumoto, Akihiro Tokushige, Mitsuru Ohishi

**Affiliations:** Department of Cardiovascular Medicine and Hypertension, Graduate School of Medical and Dental Sciences, Kagoshima University, 8-35-1 Sakuragaoka, Kagoshima, Kagoshima 890-8520 Japan

**Keywords:** Rotational atherectomy, Coronary artery disease, Coronary calcification, Percutaneous coronary intervention, Slow flow

## Abstract

Elevation of the ST segment after percutaneous coronary intervention (PCI) using rotational atherectomy (RA) for severely calcified lesions often persists after disappearance of the slow-flow phenomenon on angiography. We investigated clinical factors relevant to prolonged ST-segment elevation following RA among 152 patients with stable angina undergoing elective PCI. PCI procedures were divided into two strategies, RA without (primary RA strategy) or with (secondary RA strategy) balloon dilatation before RA. Incidence of prolonged ST-segment elevation after disappearance of slow-flow phenomenon was higher in the 56 patients with primary RA strategy (13%) than in the 96 patients with secondary RA strategy (3%, *p* = 0.039). Univariate logistic regression analysis showed levels of low-density lipoprotein cholesterol (LDL-C) (odds ratio [OR] 0.95, 95% confidence interval [CI] 0.93–0.99; *p* = 0.013), levels of triglycerides (OR 0.97, 95%CI 0.94–0.99; *p* = 0.040), and secondary RA strategy (OR 0.23, 95% CI 0.05–0.85; *p* = 0.028) were inversely associated with occurrence of prolonged ST-segment elevation following ablation. However, hemodialysis, diabetes mellitus, left-ventricular ejection fraction, lesion length ≥ 20 mm, and burr size did not show significant associations. Multivariate logistic regression analysis modeling revealed that secondary RA strategy was significantly associated with the occurrence of prolonged ST-segment elevation (Model 1: OR 0.24, 95% CI 0.05–0.95, *p* = 0.042; Model 2: OR 0.17, 95% CI 0.03–0.68, *p* = 0.018; Model 3: OR 0.21, 95% CI 0.03–0.87, *p* = 0.041) even after adjusting for levels of LDL-C and triglycerides. Secondary RA strategy may be useful to reduce the occurrence of prolonged ST-segment elevation following RA.

## Introduction

Coronary artery calcification is significantly associated with major adverse cardiac events in patients with coronary artery disease (CAD) [1, 2], and severely calcified lesions are significantly associated with poor outcomes after percutaneous coronary intervention (PCI) [1, 3, 4]. Rotational atherectomy (RA) has been widely used for severely calcified lesions in performing PCI. However, RA procedure may induce unique complications such as the slow-flow phenomenon, vessel perforation/rupture, and burr entrapment [5]. Those complications may then contribute to poor outcomes after PCI [6]. The slow-flow phenomenon is the most frequently observed complication of RA and leads to prolonged ST-segment elevation. The incidence of the slow-flow phenomenon has been reported as approximately 5–20% [7–9], although the timing of judgement (just after RA or on final angiograms) has differed among reports. Lesion length and burr-to-artery ratio are considered as determinants of slow-flow phenomenon [7], and short ablation time and gentle manipulation avoiding excessive decreases in burr rotation speed may be important to reduce the amount of debris caused by RA [5]. Intra-coronary vasodilators such as nitroprusside, nicorandil, and nitroglycerine are used to treat slow-flow phenomenon [10, 11]. However, in some cases, elevation of the ST segment persists after disappearance of the slow-flow phenomenon with the administration of vasodilators.

We investigated clinical factors related to the incidence of prolonged ST-segment elevation following ablation with RA in stable CAD patients undergoing successful PCI using RA.

## Methods

### Study design and informed consent

We investigated 152 consecutive stable CAD patients with severely calcified lesions admitted to Kagoshima University Hospital for PCI between January 2018 and August 2022 in this retrospective cohort study. This study was approved by the Research and Ethics Committee of Kagoshima University Hospital and was carried out in accordance with the ethical principles stated in the 1975 Declaration of Helsinki. All patients provided written informed consent after admission, agreeing with the use of clinical test data for scientific research by the hospital.

### Study population

Subjects comprised stable CAD patients with severely calcified lesions who underwent coronary angiography and successful PCI for clinical chest symptoms and/or myocardial ischemia, which was evaluated by fractional flow reserve or myocardial perfusion single-photon emission computed tomography. Inclusion criteria were as follows: 1) RA was required to debulk severely calcified lesions; and 2) intravascular ultrasound (IVUS) was performed. The exclusion criteria were as follows: 1) cases in which optical coherence tomography (OCT) or optical frequency domain imaging alone was used instead of IVUS; or 2) cases in which RA was performed after insertion of mechanical circulatory support. All patients were administered strong statin irrespective of dyslipidemia and dual-antiplatelet therapy (aspirin and thienopyridine: prasugrel or clopidogrel) before the procedure.

### RA procedure and IVUS

**Fig. 1:**
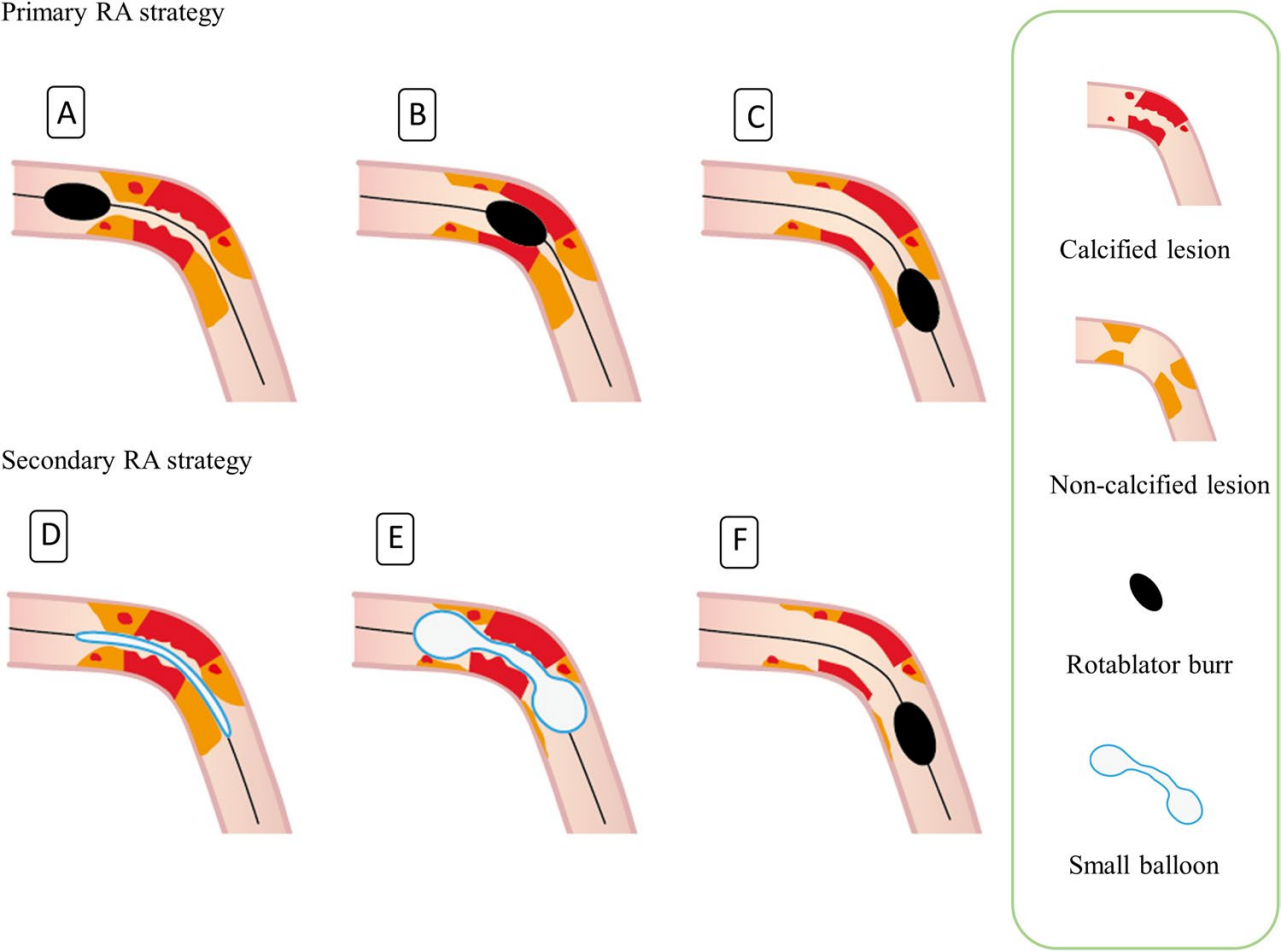
Differences in rotational atherectomy (RA) strategies. Primary RA strategy (**A**–**C**). Secondary RA strategy (**D**–**F**)

RA was performed using standard techniques, as previously described [12]. Initial rotational speed was set within the conventional range (180,000–200,000 rpm). Each run was performed with a slow pecking motion within 20 s, and excessive decreases in burr rotation speed (> 5000 rpm) were avoided as much as possible. We performed IVUS using a commercially available system (Altaview; Terumo, Tokyo, Japan) which automatically recorded at a pullback rate of 3 or 9 mm/s (30 or 10 frames/s). We assessed the grade of calcification in the target lesion by IVUS, and calcification with a calcium arch extending > 180° and calcium length > 5 mm in the target lesion was defined as moderate/severe calcification [13]. A primary RA strategy was defined as a procedure without antecedent balloon dilatation before RA (Fig. 1A–C) and secondary RA strategy was defined as a procedure with pre-dilatation using a small balloon (balloon/artery ratio ≤ 0.6 derived from IVUS measurement) (Fig. 1D–F). Primary or secondary RA strategy was performed on the basis of the operator’s judgement. In all cases, continuous intravenous infusion of nicorandil was started before the PCI procedure, and a drug cocktail (verapamil 5 mg, nitroglycerin 5 mg, heparin 10000 unit, and saline 500 ml) was infused into a targeted coronary artery through the RA catheter to prevent slow flow. Angiography was performed to confirm whether slow flow occurred immediately after RA in every case. Slow flow was defined as thrombolysis in myocardial infarction (TIMI) flow grade ≤ 2 without lesions leading to delayed filling of distal vessels. The burr-to-artery ratio was defined as the burr size divided by the reference diameter derived from IVUS measurements. In all cases, monitoring by 12-lead electrocardiography was performed during PCI and recorded at the end of PCI.

### Assessments and definitions of clinical characteristics

Laboratory values were obtained on admission before PCI. Left-ventricular ejection fraction (LVEF) was obtained from the official echocardiography report within 1 month before RA. Strong statins were defined as doses of atorvastatin ≥ 10 mg/day, rosuvastatin ≥ 2.5 mg/day, or pitavastatin ≥ 2 mg/day, based on PATROL trial showing the efficacy and safety of statin use in Japanese populations [14].

### Clinical outcomes

Clinical outcomes were retrospectively collected from our hospital records. Prolonged ST-segment elevation after RA was defined continued ST-segment elevation despite administration of intra-coronary vasodilators such as nitroprusside after the disappearance of slow-flow phenomenon without lesions leading to delayed filling of distal vessels. Peri-procedural myocardial infarction, including side-branch occlusion due to stenting, was defined as an elevation of creatinine kinase levels at least threefold above the normal upper limit within 48 h after PCI [1, 15].

### Statistical analysis

Descriptive statistics are presented as frequency (percentage) for categorical variables and mean ± standard deviation or median and interquartile range (IQR) for continuous variables. Fisher’s exact test was used to compare the incidence of categorical variables, which were expressed as frequency and percentage. A logistic regression analysis was used to assess factors associated with prolonged ST-segment elevation after RA, reporting odds ratios (ORs) and 95% confidence intervals (CIs). Variables showing values of *p* < 0.05 on univariate analysis were entered into multivariate analysis. In addition, logistic regression modeling was performed to assess ORs for prolonged ST-segment elevation after RA. To evaluate the robustness of our results and assess the impact of confounding variables, we added the confounding variables to our model for each of the multiple pairs and compared the adjusted odds ratios. *P* values < 0.05 were considered statistically significant and all analyses were conducted using SAS software (JMP version 14.0).

## Results

### Baseline patient characteristics

The baseline clinical characteristics of patients are shown in Table 1. Median age was 71 years (IQR 66–80 years), and 108 patients (71%) were male. The primary RA strategy was applied in 56 patients (37%) and the secondary RA strategy in the remaining 96 patients (63%). In secondary RA strategy group, fasting plasma glucose level [median, 116 interquartile range (IQR) 97–160 mg/dL vs. 104 IQR 87–129, *p* = 0.031], using of drug-coated balloon (29% vs. 45%; *p* = 0.036), and 2-burr usage (21% vs. 36%; *p* = 0.038) were higher than those of primary RA strategy group. Age, diabetes mellitus, hemodialysis, LVEF, lesion length ≥ 20 mm, and burr size did not show significant differences between the primary RA strategy and secondary RA strategy groups.

**Table 1:** Baseline characteristics of study participants

Variables	Overall (*n* = 152)	Primary RA strategy (*n* = 56)	Secondary RA strategy (*n* = 96)	*p* value
Age, years	71 [66, 80]	69 [64, 79]	72 [67, 81]	0.112
Sex (male), n (%)	108 (71)	40 (71)	68 (71)	1.000
BMI, kg/m^2^	23.3 [21.0, 26.1]	24.1 [21.4, 26.7]	23.2 [20.9, 25.2]	0.129
Risk factors, *n* (%)				
Hypertension	137 (90)	50 (89)	87 (91)	0.785
Diabetes mellitus	92 (61)	33 (59)	59 (61)	0.864
Dyslipidemia	108 (71)	39 (70)	69 (72)	0.853
Current smoker	31 (21)	14 (25)	17 (18)	0.305
Hemodialysis	46 (30)	17 (30)	29 (30)	1.000
CKD	108 (71)	43 (77)	65 (68)	0.269
Medication, *n* (%)				
Calcium-channel blocker	72 (47)	24 (43)	48 (50)	0.406
ACEI	18 (12)	8 (14)	10 (10)	0.604
ARB	66 (43)	23 (41)	43 (45)	0.735
Beta-blocker	68 (45)	26 (46)	42 (44)	0.866
Statin	152 (100)	56 (100)	96 (100)	NA
Laboratory data				
LDL-C, mg/dL	71 [56, 95]	67 [54, 82]	76 [57, 97]	0.089
HDL-C, mg/dL	51 [43,60]	51 [43,60]	51 [41,60]	0.636
Triglycerides, mg/dL	93 [74, 119]	96 [80, 119]	92 [72, 121]	0.316
FPG, mg/dL	113 [94,153]	104 [87,129]	116 [97,160]	0.031
Hemoglobin g/dL	12.1 [10.6, 13.5]	12.2 [10.5, 13.4]	12.0 [10.6, 13.6]	0.852
eGFR, mL/min/1.73 m^2^	40.7 [8.9,58.7]	37.1 [7.9,61.2]	42.4 [9.6,58.6]	0.305
LVEF, %	58.6 [45.0, 67.8]	63.3 [50.5, 69.0]	56.2 [45.0, 66.4]	0.062
Target lesion, *n* (%)				
LAD	89 (59)	35 (63)	54 (56)	0.497
RCA	37 (24)	9 (16)	28 (29)	0.080
LCX	24 (16)	11 (20)	13 (14)	0.360
LMT	2 (1)	1 (2)	1 (1)	1.000
Lesion length, *n* (%)				
≥ 20 mm	113 (74)	38 (68)	75 (78)	0.115
< 20 mm	39 (26)	18 (32)	21 (22)	0.943
Final device, *n* (%)				
DES	93 (61)	40 (71)	53 (55)	0.985
DCB	59 (39)	16 (29)	43 (45)	0.036
Max. burr size, *n* (%)				
1.25 mm	13 (9)	5 (9)	8 (8)	1.000
1.5 mm	83 (56)	29 (52)	54 (56)	0.616
1.75 mm	49 (32)	17 (30)	32 (33)	0.723
2.0 mm	7 (5)	5 (9)	2 (2)	0.101
Number of burrs used, *n* (%)				
1	105 (70)	44 (79)	61 (64)	0.984
2	47 (31)	12 (21)	35 (36)	0.038

Values are shown as median with interquartile range or number and percentage

*ACEI* angiotensin-converting enzyme inhibitor, *ARB* angiotensin II receptor blocker, *BMI* body mass index, *CKD* chronic kidney disease, *DCB* drug-coated balloon, *DES* drug-eluting stent, *eGFR* estimated glomerular filtration rate, *FPG* fasting plasma glucose, *HDL-C* high-density lipoprotein cholesterol, *LAD* left anterior descending artery, *LCX* left circumflex artery, *LDL-C* low-density lipoprotein cholesterol, *LMT* left main trunk, *LVEF* left-ventricular ejection fraction, *RA* rotational atherectomy, *RCA* right coronary artery

### Clinical complication during PCI procedure and outcomes after PCI

Major complications induced by RA were not encountered in any cases (Table 2). Slow flow (final coronary angiography TIMI ≤ 2) was only seen in one case (2%), in a patient treated using the primary RA strategy. This case involved debulking by RA for in-stent restenosis with severe diffuse calcification. Prolonged ST-segment elevation following ablation was observed in ten cases (7%) in total, with patients treated using the primary RA strategy showing a higher incidence (13%) than patients treated using the secondary RA strategy (3%, *p* = 0.039).

**Table 2:** Major complication of rotational atherectomy (RA)

Variable, *n* (%)	Primary RA strategy	Secondary RA strategy	*p* value
Perforation	0 (0)	0 (0)	NA
Rupture	0 (0)	0 (0)	NA
Burr entrapment	0 (0)	0 (0)	NA
Disconnection of burr	0 (0)	0 (0)	NA
Stent deformation	0 (0)	0 (0)	NA
Slow flow	1 (2)	0 (0)	0.368
Cardiac shock	0 (0)	0 (0)	NA
Prolonged ST-segment elevation	7 (13)	3 (3)	0.039
Procedure-related myocardial infarction	3 (5)	3 (3)	0.670

*NA* not available

### Baseline systemic factors associated with prolonged ST-segment elevation following RA

Univariate logistic analysis was performed to investigate associations of baseline characteristics (including lesion characteristics and PCI procedure) with the occurrence of prolonged ST-segment elevation following ablation. In univariate analysis, levels of low-density lipoprotein cholesterol (LDL-C) (OR 0.95, 95% CI 0.93–0.99; *p* = 0.013), levels of triglyceride (OR 0.97, 95% CI 0.94–0.99; *p* = 0.040), and secondary RA strategy (OR 0.23, 95% CI 0.05–0.85; *p* = 0.028) were inversely associated with the occurrence of prolonged ST-segment elevation following RA (Table 3).

**Table 3:** Univariate logistic regression analysis for occurrence of prolonged ST-segment elevation following ablation

Variable	OR	95% CI	*p* value
Age, years	1.05	0.98–1.13	0.156
Sex (male)	0.58	0.16–2.40	0.429
BMI, kg/m^2^	0.96	0.78–1.12	0.636
Risk factors			
Hypertension	0.98	0.16–18.8	0.989
Diabetes mellitus	1.56	0.41–7.49	0.529
Dyslipidemia	3.90	0.70–73.19	0.203
Current smoker	0.41	0.02–2.31	0.408
Hemodialysis	0.98	0.20–3.73	0.985
CKD	1.67	0.39–11.40	0.526
Medication			
Calcium-channel blocker	1.01	0.25–4.81	0.901
ACEI	0.82	0.04–4.76	0.852
ARB	1.33	0.35–4.97	0.665
Beta-blocker	0.50	0.11–1.91	0.340
Laboratory data			
LDL-C	0.95	0.93–0.99	0.013
HDL-C	0.99	0.94–1.04	0.797
Triglycerides	0.97	0.94–0.99	0.040
FPG	1.00	0.99–1.01	0.410
eGFR	0.99	0.97–1.02	0.812
LVEF	0.99	0.96–1.05	0.986
Target lesion			
LAD	1.71	0.45–8.17	0.452
RCA	1.36	0.28–5.19	0.667
LCX	NA		
LMT	NA		
Lesion length			
≥ 20 mm	1.26	0.26–4.80	0.754
Final device			
DES	0.95	0.26–3.85	0.937
DCB	1.05	0.26–3.85	0.937
Max. burr size			
1.25 mm	1.20	0.06–7.26	0.866
1.5 mm	1.27	0.35–5.13	0.724
1.75 mm	0.51	0.07–2.11	0.400
2.0 mm	2.52	0.13–17.1	0.415
Number of burrs used			
2	2.38	0.63–8.98	0.188
Strategy			
Primary RA strategy	4.42	1.18–21.25	0.037
Secondary RA strategy	0.23	0.05–0.85	0.028

*CI* confidence interval, *OR* odds ratio, *ACEI* angiotensin-converting enzyme inhibitor, *ARB* angiotensin II receptor blocker, *BMI* body mass index, *CKD* chronic kidney disease, *DCB* drug-coated balloon, *DES* drug-eluting stent, *eGFR* estimated glomerular filtration rate, *FPG* fasting plasma glucose, *HDL-C* high-density lipoprotein cholesterol, *LAD* left anterior descending artery, *LCX* left circumflex artery, *LDL-C* low-density lipoprotein cholesterol, *LMT* left main trunk, *LVEF* left-ventricular ejection fraction, *RA* rotational atherectomy, *RCA* right coronary artery, *NA* not available

### Predictive value of prolonged ST-segment elevation following RA

Multivariate logistic regression analysis modeling for the incidence of prolonged ST-segment elevation revealed that the secondary RA strategy was significantly associated with the occurrence of prolonged ST-segment elevation following ablation in all three models (Model 1: OR 0.24, 95% CI 0.05–0.95, *p* = 0.042; Model 2: OR 0.17, 95% CI 0.03–0.68, *p* = 0.018; Model 3: OR 0.21, 95% CI 0.03–0.87, *p* = 0.041) even after adjusting for relevant factors such as levels of LDL-C and triglyceride (Table 4). In addition, LDL-C (Model 1: OR 0.96, 95% CI 0.92–0.99, *p* = 0.020) and triglyceride (Model 2: OR 0.96, 95% CI 0.94–0.99, *p* = 0.025) correlated significantly with prolonged ST-segment elevation in Models 1 and 2. However, LDL-C and triglycerides did not reach the level of a significant independent association with prolonged ST-segment elevation in Model 3.

**Table 4:** Predictive values for occurrence of prolonged ST-segment elevation following ablation as determined by multivariate logistic regression analysis

Variables	Model 1			Model 2			Model 3		
	OR	95% CI	*p* value	OR	95% CI	*p* value	OR	95% CI	*p* value
Secondary RA strategy	0.24	0.05–0.95	0.042	0.17	0.03–0.68	0.018	0.21	0.03–0.87	0.041
LDL-C	0.96	0.92–0.99	0.020	–	–	–	0.97	0.92–0.99	0.079
Triglyceride	–	–	–	0.96	0.94–0.99	0.025	0.98	0.95–1.00	0.104

Model 1, adjusted for LDL-C; Model 2, adjusted for triglycerides; Model 3, adjusted for Model 1 variables plus triglycerides

*ACEI* angiotensin-converting enzyme inhibitor, *ARB* angiotensin II receptor blocker, *BMI* body mass index, *CKD* chronic kidney disease, *DCB* drug-coated balloon, *DES* drug-eluting stent, *eGFR* estimated glomerular filtration rate, *FPG* fasting plasma glucose, *HDL-C* high-density lipoprotein cholesterol, *LAD* left anterior descending artery, *LCX* left circumflex artery, *LDL-C* low-density lipoprotein cholesterol, *LMT* left main trunk, *LVEF* left-ventricular ejection fraction, *RA* rotational atherectomy, *RCA* right coronary artery, *CI* confidence interval, *OR* odds ratio

## Discussion

In this study, we showed that: (1) in patients with stable CAD undergoing PCI with RA, the secondary RA strategy correlated independently with the occurrence of prolonged ST-segment elevation after disappearance of slow-flow phenomenon on angiography; and (2) rates of complications related to RA procedures did not differ significantly between primary and secondary RA strategies.

The slow-flow phenomenon is frequently observed during PCI, particularly in acute coronary syndrome, and is well known to be significantly associated with thrombus or lipid-rich plaques in the target lesions, both of which are prone to induce distal coronary artery embolization [7, 16]. Furthermore, slow-flow phenomenon affects the prognosis after PCI. When performing PCI with RA for calcified lesions, the slow-flow phenomenon is frequently encountered [12]. Previous studies have reported that lesion length as measured angiographically was associated with the occurrence of slow flow after RA [7, 17] and that the arc of calcification at the minimal lumen area derived from IVUS represents a new factor associated with slow flow after RA [18]. Furthermore, Sakakura et al. showed that IVUS cross-ability was also significantly associated with slow flow after RA [19]. A clinical expert consensus document on rotational atherectomy from the Japanese Association of Cardiovascular Intervention and Therapeutics reported risk factors and treatment of the slow-flow phenomenon. This document reported lesion length and burr-to-artery ratio as determinants of slow flow [7], and noted that appropriate burr size, short ablation time, and gentle manipulation avoiding excessive decreases in burr rotation speed would be important to minimize the amount of debris caused by RA. Moreover, intra-coronary vasodilators such as nitroprusside, nicorandil, and nitroglycerine are useful for the treatment of slow flow [10]. However, ST-segment elevation following RA often persists even after disappearance of slow-flow phenomenon on angiography following administration of vasodilators. We therefore investigated clinical factors in the incidence of prolonged ST-segment elevation following RA in patients with stable CAD who underwent successful PCI using RA.

A previous study reported that primary RA strategy was inversely associated with slow flow, whereas the secondary RA strategy was positively associated with slow flow [12]. The present findings suggest that the primary RA strategy would be advantageous for slow flow by not inducing coronary dissection or intimal hematoma, because antecedent balloon dilatation was not performed before RA. Although no data concerning balloon size before RA were reported in the previous study, we performed all pre-dilatations using small balloons (balloon/artery ratio ≤ 0.6) within nominal pressure in the secondary RA strategy in the present study. While this procedure may minimize the incidence of coronary dissection or intimal hematoma, we were unable to confirm this possibility. Our study focused on the prolonged ST-segment elevation after recovery from slow-flow phenomenon, and only the second RA strategy had a protective effect on prolonged ST-segment elevation. Therefore, coronary dissection or intimal hematoma induced by pre-ballooning before RA may not be an important factor for prolonged ST-segment elevation.

The mechanism of prolonged ST-segment elevation after disappearance of slow flow may be multifactorial. One potential mechanism is the amount of debris from calcified lesions as suggested in the coronary circulation. In severely calcified stenotic lesions, non-/mildly calcified plaques are often continuously interspersed in the proximal and distal portions (Fig. 1A). When RA is performed on such lesions under the primary RA strategy, ablation of the less calcified plaques is inevitable and may lead to large amounts of debris from the lesions (Fig. 1C). Conversely, in the second RA strategy, dilating the lesion with a small-diameter balloon before RA can create an easy pathway to severely calcified lesions for the rotablator burr due to the expansion of less calcified plaques (Fig. 1E). We can avoid the impact of ablation on less calcified lesions and achieve effective debulking only for severely calcified lesions. In addition, the time required to achieve ablation can be minimized. Consequently, the second RA strategy may be able to minimize the debris from target lesions compared with the primary RA strategy and may thus have protective effects on prolonged ST-segment elevation.

Lipid-lowering therapy is an important strategy in the primary and secondary prevention of CAD [20, 21]. Many clinical trials using statins have revealed that lowering LDL-C was significantly associated with substantial reductions in major cardiovascular events [22–25]. All patients were, therefore, administered a strong statin regardless of dyslipidemia. In our study, LDL-C and triglycerides were negatively associated with prolonged ST-segment elevation after RA calcification, although neither reached the level of a significant association in Model 3. In the case of high LDL-C and triglyceride levels, triglyceride-rich lipoprotein includes very low-density lipoprotein, which could contribute to the pathogenesis of atherosclerosis through increased expression of proinflammatory cytokines and induction of endothelial cell apoptosis [26–30], while in the case of low LDL-C and triglyceride levels, statin therapy could contribute to plaque stabilization and increased calcium deposits due to the anti-inflammatory effects of statins [31]. As a result, patients with lower LDL-C and triglyceride levels may have more calcified plaques, and the slow-flow phenomenon and/or prolonged ST-segment elevation following RA may tend to occur.

### Limitations

There are several limitations in the present study. First, this retrospective study of a single center involved a relatively small cohort of patients. Second, we did not analyze the IVUS parameters associated with lipid components that were recognized as factors in slow flow in this study. Third, there were a small number of cases with OCT-guided RA in the current study period, and the definition of severe calcification differs between OCT and IVUS. Furthermore, the burr-to-artery ratio was calculated using the reference diameter derived from IVUS. Therefore, the cases with OCT-guided RA were excluded in this study. Fourth, although all patients were administered a strong statin, the dose and specific agent were not standardized. Finally, as this study is retrospective, inherited limitations such as the potential for unmeasured confounding cannot be ruled out. Prospective clinical investigations of larger populations are necessary to clarify the effect of secondary RA strategy on the incidence of prolonged ST-segment elevation.

## Conclusion

The secondary RA strategy appears to represent the safest procedure for debulking severely calcified lesions and may be useful to reduce the occurrence of prolonged ST-segment elevation after RA.

## Data Availability

The datasets used and/or analyzed during the current study are available from the corresponding author on reasonable request.

## Abbreviations

BMI: Body mass index
CAD: Coronary artery disease
CI: Confidence interval
IQR: Interquartile range
IVUS: Intravascular ultrasound
LDL-C: Low-density lipoprotein cholesterol
LVEF: Left-ventricular ejection fraction
OR: Odds ratio
PCI: Percutaneous coronary intervention
RA: Rotational atherectomy
TIMI: Thrombolysis in myocardial infarction
